# Biology of Ageing and Role of Dietary Antioxidants

**DOI:** 10.1155/2014/831841

**Published:** 2014-04-03

**Authors:** Cheng Peng, Xiaobo Wang, Jingnan Chen, Rui Jiao, Lijun Wang, Yuk Man Li, Yuanyuan Zuo, Yuwei Liu, Lin Lei, Ka Ying Ma, Yu Huang, Zhen-Yu Chen

**Affiliations:** ^1^Food & Nutritional Sciences Programme, School of Life Sciences, The Chinese University of Hong Kong, Shatin, Hong Kong; ^2^Lipids Technology and Engineering, College of Food Science and Technology, Henan University of Technology, Henan, China; ^3^Department of Food Science and Engineering, Jinan University, Guangzhou, China; ^4^School of Biomedical Sciences, The Chinese University of Hong Kong, Shatin, Hong Kong

## Abstract

Interest in relationship between diet and ageing is growing. Research has shown that dietary calorie restriction and some antioxidants extend lifespan in various ageing models. On the one hand, oxygen is essential to aerobic organisms because it is a final electron acceptor in mitochondria. On the other hand, oxygen is harmful because it can continuously generate reactive oxygen species (ROS), which are believed to be the factors causing ageing of an organism. To remove these ROS in cells, aerobic organisms possess an antioxidant defense system which consists of a series of enzymes, namely, superoxide dismutase (SOD), catalase (CAT), glutathione peroxidase (GPx), and glutathione reductase (GR). In addition, dietary antioxidants including ascorbic acid, vitamin A, vitamin C, **α**-tocopherol, and plant flavonoids are also able to scavenge ROS in cells and therefore theoretically can extend the lifespan of organisms. In this connection, various antioxidants including tea catechins, theaflavins, apple polyphenols, black rice anthocyanins, and blueberry polyphenols have been shown to be capable of extending the lifespan of fruit flies. The purpose of this review is to brief the literature on modern biological theories of ageing and role of dietary antioxidants in ageing as well as underlying mechanisms by which antioxidants can prolong the lifespan with focus on fruit flies as an model.

## 1. Introduction

Our understanding on aging is still quite limited. As a complex biological process, aging involves a variety of factors. On the one hand, the variation of average lifespan from different regions is believed to be due to the differences in not only genes but also environmental conditions and eating habits. On the other hand, most organisms actually die from age-related diseases rather than aging itself. In modern society, aging-related neurodegenerative diseases have been a rising lethal threat to human beings. WHO has promoted the concept of “healthy lifespan,” aiming to increase the ratio of healthy to total lifespan.

The first documented study on aging was conducted in 1532 by Muhammad in his book “Ainul Hayat.” Almost 5 centuries have passed, and the mechanism and cause of aging are still not clear. In order to increase both average and maximum lifespans as well as to decrease the occurrence of age-related diseases, the mechanism behind aging needs to be explored at molecular level. Recently, extensive research has attempted to identify mechanisms underlying the links between diets and health. This review will summarize the biological theories of ageing and review the research on role of dietary antioxidants in delaying the aging.

## 2. Aging Theories

### 2.1. Stochastic Theories of Aging (STA)

STA proposes that aging is the result of inevitable small random changes that accumulate with time and the failure of repairing stochastic damages in cells. The precursor of this concept is the wear and tear theory, initially proposed by August Weismann, who believed that the aging was due to constantly exposed to wounds, infections, and injuries and also from time to time, consuming excessive fat, sugar, and receiving undue UV lights or outsourced stresses. The accumulated damages would cause minor damages to cells and tissues, contributing to the age-related decline of organ functional efficiency. It has been revealed that animals that are raised in protected environment and do not suffer from those minor exogenous insults, still age. Later on, the theory is modified by incorporating the failure of repair hypothesis. For example, somatic mutation postulates that aging is due to alterations of chromosome number or formations of lesions in existing chromosomes, caused by accumulation of stochastic genetic mutations. Evidence gathered by Hart and Setlow [1] helps to develop the theory of DNA damage and repair. It is claimed that DNA damage contributes to aging process because there is a positive correlation between DNA repair capacity and lifespan. However, nowadays STA is no longer regarded to be the sole potential candidates for the explanation of aging. As a promising modified successor, free radical theory has been becoming one of the most widely accepted aging mechanism hypotheses.

### 2.2. Free Radical Theory of Ageing (FRTA)

FRTA was first proposed by Harman [2], stating that aging is due to accumulation of oxidative damages to tissues and organs caused by free radicals. It has been considered as one of the major theories providing a testable biological mechanism for aging process. Free radicals are any substances with unpaired electrons and readily react with healthy molecules in a destructive way. They can be produced in large quantities in cells by different mechanisms, such as exposure to oxygen, radiation, or environmental toxins, for example, pesticide and herbicide. The three major stages of free radical reactions are initiation, propagation, and termination. No matter how it is initiated, once formed, the free radicals can propagate itself indefinitely in the presence of oxygen until those radicals reach a high concentration to react with each other and produce a nonradical species [3].

Reactive Oxygen Species (ROS), the most abundant free radicals in cells, cover a wider range. Generally speaking, any highly reactive molecules containing oxygen can be classified into this category. ROS are unavoidable products during normal intracellular metabolism. They actually play essential roles in cell differentiation, proliferation, and host defense response [4]. However, their bad reputations are definitely overwhelming. Various cell components are believed to be damaged by oxygen-derived free radicals, of which lipid peroxidation, DNA damage, and protein oxidation are probably the most critical.

ROS can cause the lipid oxidation in cells. Polyunsaturated fatty acids, the main component of cell membranes, are vulnerable to free radical attack because they contain such multiple double bonds, which possess extremely reactive hydrogen atoms. As a result, the structure is susceptible to be attacked by free radicals, especially hydroxyl radicals, which will lead to the destruction of cell membrane permeability, and eventually the cellular dysfunction [5].

ROS can also damage the DNA. The ROS-induced DNA damage mainly includes strand break, cross-linking, base hydroxylation, and base excision. The induction of those DNA damages will result in mutagenesis and consequently transformation, especially if combined with a deficient apoptotic pathway [6, 7].

ROS can also lead to the oxidation of proteins in vivo. The proteins in cells are also believed to be the main targets of free radicals. Aromatic amino acids, cysteine, and disulphide bonds are susceptible to the attack of free radicals, which will lead to protein denaturation and enzyme inactivation [5]. Furthermore, the reactive protein derivatives generated might act as intermediates to induce propagation of oxidative damages to other cell components [8].

**Figure 1:**
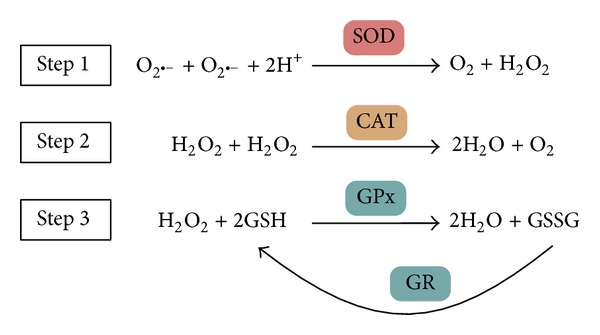
Main enzymatic antioxidant defense system in vivo and their reactions on scavenging free radicals and hydrogen oxide. SOD, superoxide dismutase; CAT, catalase; GPx, glutathione peroxidase; and GR, glutathione reductase.

Two main antioxidant systems, namely, enzymatic antioxidants and nonenzymatic ones, act systematically to scavenge the free radicals [9]. The enzymatic antioxidant system consists of superoxide dismutase (SOD), catalase (CAT), glutathione peroxidase (GPx), and glutathione reductase (GR) (Figure 1). This system is the main defense system against ROS in vivo. There are two major types of SOD. One is CuZnSOD (SOD1), which mainly exist in cytoplasm, with copper and zinc being present in the active site. The other one is MnSOD (SOD2), locating in mitochondrial matrix, with manganese being present in the active site. They can catalyze the reaction to decompose superoxide anion radicals into H_2_O_2_, which will then be converted to water and oxygen by CAT or GPx. CAT is one of the most efficient redox enzymes, with iron being present in its active site, mainly found in peroxisome [10]. It can catalyze the conversion of H_2_O_2_ into water and oxygen. Otherwise, H_2_O_2_ would be converted to hydroxyl radical, one of the most active and harmful radicals to living cells. GPx is a selenium-containing enzyme, protecting cells and tissues from oxidative damage by removing H_2_O_2_ with the oxidization of glutathione. On the other hand, GR can convert the oxidized glutathione to its reduced form. However, the contribution of GPx in insects including fruit flies is relatively low [11].

The nonenzymatic antioxidants system serves as the second defense system against the free radicals. Nonenzymatic antioxidants can not only provide direct protection against oxidative damages but also more importantly enhance the function of endogenous enzymatic antioxidants by synergistically scavenging the reactive free radicals [12]. Vitamins C and E are the most renowned antioxidants in this category. However, recent study revealed that under certain circumstances, they might function as prooxidants [13]. In addition to vitamins, there are many small molecules which serve as nonenzymatic antioxidants, such as phenolic, flavonoids, and carotenoids naturally present in foods. They can be obtained from daily diets, belonging to a group of food-derived phytochemicals called nutraceuticals [14, 15].

### 2.3. Mitochondrial Decline Theory of Aging (MDTA)

MDTA has for so long been proposed to explain the aging process [16, 17]. Mitochondrial respiratory capacity declines with aging. Cytochrome c oxidase (CcO), the terminal oxidoreductase of mitochondrial electron transport chain (ETC), is consistently reported to decline in both aged invertebrates and vertebrates [18, 19]. Especially, its subunits III and VIb are significantly reduced in aging flies [20]. It has been reported that CcO deficiency would result in reduction of total ETC activity due to the increased production of either superoxide anion radicals or hydrogen peroxide in mitochondria. Therefore, there are solid connections between MDTA and FRTA. Theoretically speaking, enhancing antioxidant defense system will not only lead to reduced amount of free radicals but also ameliorate the functional decline of mitochondria.

### 2.4. Decline Theory of Ubiquitin Proteasomal System (UPS)

Protein misfolding and aggregation are essential factors, contributing significantly to aging process and especially to the formation and development of neurodegenerative diseases, such as Parkinson's disease (PD) and Alzheimer's disease (AD) [21]. They can be cleared mainly by UPS [22, 23]. It is reported that age-related decline is associated with the lower activity of the 26S proteasome. Thus, maintenance of the 26S proteasome activity with age is vital for promoting longevity. The 26S proteasome is a complex of the 20S core chamber attached to two 19S caps on each end. The 20S proteasome itself cannot degrade multiubiquitinated proteins since the pores leading into the catalytic chamber are closed. The opening of the gates is triggered by the 19S attached to the ends of the 20S core chamber [24, 25].

Rpn11 is one lid component of the multiple subunits making up the 19S, which can be divided into two subcomplexes, that is, the base and lid. It is reported that knock down of Rpn11 will reduce 26S proteasome activity, leading to increased age-related accumulation of ubiquitinated proteins and shorter lifespan. On the contrary, overexpressing Rpn11 can reduce age-related accumulation of ubiquitinated proteins and thus extends lifespan [26].

### 2.5. Genetic Theory of Ageing

The genetic theory of ageing states that longevity is largely determined by the genes. As one of the most complicated biological processes, aging involves factors covering a wide range from genetic to environmental ones. Single gene mutation has been proved to be one of the most useful techniques to understand aging mechanisms at molecular level. Previous studies in* C. elegans*,* Drosophila*, and rodents have revealed dozens of genes, whose mutation would lead to extended lifespan. Those selected genes are named as longevity determined genes [27, 28] (Table 1).

In* Drosophila*, single P-element insertion mutation lines can be easily generated [29] and the newly inserted locus could be identified by flanking sequence of the inserted transposon [30]. Lin et al. [31] reported that a P-element insertion was identified with an extra 35% longer lifespan, compared to wild type flies (Table 1). At the same time, they found that these* methuselah* (Mth) mutant flies showed higher resistance to various stresses, such as high temperature, starvation, and paraquat [31]. Mth protein belongs to class B of G protein-coupled receptors (GPCRs), a protein family with their iconic, large ligand-binding N-terminal extracellular domains, playing a key role in intracellular signal transduction [32, 33]. To date, the specific function of Mth is still unknown. It has been demonstrated that flies expressing a Mth antagonist peptide live significantly longer [34]. Humans have homologous gene to Mth (APG1), which could be a promising candidate for development of antiageing drugs [32].

Many other genes may be involved in the process of aging. In this connection, it has been shown that decreased expression of Indy gene in fly and worm extends longevity [35]. Indy gene encodes a transporter of Krebs cycle intermediates with the highest rate of uptake for citrate. It is known that cytosolic citrate has a role in energy regulation by affecting fatty acid synthesis and glycolysis [35]. It has been also found that chico gene, encoding an insulin receptor substrate that functions in an insulin/insulin-like growth factor (IGF) signaling pathway, has a role in aging as mutation of chico extends fruit fly median lifespan by up to 48% in homozygotes and 36% in heterozygotes [36]. Some evidence suggests that the fat body in* Drosophila* acts as a nutrient sensor, which uses TOR signaling to generate a humoral signal that modulates insulin signaling and growth in peripheral tissues. Modulation on its activity of gene in the TOR pathway leads to a longer lifespan [37]. Recent work suggests that sirtuins, encoding a conserved family of nicotinamide adenine dinucleotide (NAD+)-dependent protein deacetylases, have been also shown to regulate lifespan in many model organisms including yeast and mice by modulating ROS levels notably during a calorie restriction [38].

## 3. *Drosophila* and Other Models in Aging Research

It is critical to conduct a study on proper models in order to elucidate the aging mechanisms more thoroughly. Studies on humans are most straightforward. However, the duration of human aging is a limiting factor since researchers themselves also, at the same time, go through the same process. Meanwhile, ethical issues also block many research experiments on human beings. Therefore, it turns to laboratory model systems and then tries to extrapolate laboratory data to clinical value. The selection of models is diverse and under debate [39, 40]. In general, the mainstream model systems to conduct the aging study include cells, yeast (*Saccharomyces cerevisiae*), roundworms (*Caenorhabditis elegans*), fruit flies (*Drosophila melanogaster*), mice (*Mus musculus*), and rats (*Rattus norvegicus*).

Human cells are one of the major model systems in studying aging mechanisms. Researchers can easily focus on human biology when carrying out experiments on human cells. Nevertheless, in vitro data might not be always consistent with in vivo one. Meanwhile, the most widely employed parameters for cellular models on aging study are cell proliferation and stress resistance. However, the correlation of those factors with organismal aging is still under serious debate [41, 42].

Nonmammalian model systems, such as yeast, roundworms, and fruit flies, share a large number of key biological pathways with humans [43], though their physiology and phenotypes are way from alike with mammals. Meanwhile, aging researches are always based on statistical analysis and comparison at the population level. Nonmammalians models are comparatively easier and cheaper to manipulate in large numbers. On the other hand, aging is a complex biological process, involving too many factors at the same time. Nevertheless, it is reasonable and practical to conduct assays on relatively simpler systems first to observe more direct and immediate response after certain treatment. Actually, many genes and signal pathways modulating ageing process have already been identified in yeast [44], worms [45], and fruit flies [46], which serve as basis for further understanding human aging mechanisms.

As to the mammal systems, such as mice and rats, their physiology and daily activities are more parallel to humans, compared to those nonmammalian models. At the same time, as the mainstream animals employed in laboratory for decades, the related experiment protocols are quite mature and stable. However, there is still no solid evidence indicating that those rodents age for the same causes and mechanisms as humans [39]. Therefore, if a particular age-related mechanism is investigated, simpler and easier nonmammalian models might be more preferred choice.

Last but not least, nonhuman primates are recently regarded as a potential alternative for human aging studies [47]. It is claimed that Rhesus monkeys (*Macaca mulatta*) share about 90% of their genome with human beings [48]. In addition, age-related changes in neurological structure and function of monkeys also share great similarity with those of humans [49, 50]. Nevertheless, the actual employment of this nonprimate model in aging study is still quite rare for the following reasons. First of all, those primates have comparatively long lifespan, which is a practical problem for laboratory manipulation. Secondly, the costs to conduct experiments on those animals are relatively high [47]. Thirdly, a serious issue is the moral concern by animal rights groups.

*Drosophila* model has been widely used for biological researches, especially in the field of genetics and developmental biology. In light of the study on genetics of longevity in fruit flies, specific genes regulating lifespan have been revealed during the past decades, which involved in stress response, antioxidant system, insulin signaling pathway, and TOR pathway. It is reported that SOD or CAT mutant flies (partially knock out either SOD or CAT genes) will lead to much shorter lifespans along with a greater sensitivity to oxidative stress [51, 52]. On the contrary, transgenic flies with additional copies of CAT and SOD show median lifespan increase ranging from 6% to 33%, overexpression of SOD increases mean lifespan up to 40% [53–55].

Studies on the relation between diet supplements and lifespan of fruit flies have been continuously producing inspiring results. Experiments conducted by Bonilla et al. [56, 57] demonstrated that melatonin in diet could significantly increase the lifetime and the resistance to paraquat challenge in* Drosophila*. Similarly, resveratrol had been proved to be effective in lifespan extension in fruit flies by Bauer et al. [58] and Wood et al. [59]. We had demonstrated that green tea and broccoli could extend the median lifespan of fruit flies [60, 61].

Serving as an efficient model in aging research for decades, fruit flies possess unique advantages over other organism models. First, fruit flies and humans share many conserved physiological pathways, such as superoxide metabolism, insulin-like signaling, many of which have been proposed as vital elements for ageing regulation [43]. Second, more than 70% of known disease-causing genes in humans are conserved in fruit flies and 50% of fly protein sequences have mammalian homologs [62, 63]. Third, the technique of genetic manipulation in* Drosophila* is now quite mature. There are a wide variety of transgenic flies available, which simplify the exploration for the targets [64]. Meanwhile, fly strains with longer life span are reported to have no reduction in metabolic rate [65]. Fourth,* Drosophila* have complex nervous system with a relatively weak blood-brain barrier, which makes it a suitable model system for screening and evaluation of effects of drugs and functional compounds on neurodegenerative diseases [66, 67]. Fifth, fruit flies are comparatively easier and cheaper to maintain in large numbers, which is essential for a cohort study. The short life cycle, tiny body size, high fecundity, and known sequence of full genome make it an ideal model for aging research at population level [68]. In addition, the effects of diet supplements on aging of fruit flies have been investigated, providing promising results in the last 20 years, which not only construct practical bench methods to do related analysis, but also package powerful statistical protocols to systematically estimate and assess the effects of certain supplement compounds on aging [69]. By and large,* D. melanogaster* model is more than simple and valid to be employed in the study of universal aging mechanisms.

## 4. Energy Restriction (ER) Prolongs the Lifespan

ER is to reduce moderately nutrient availability without malnutrition. ER has been shown to extend the lifespan of diverse organisms including rodents, yeast,* Drosophila,* and* C. elegans* [40, 70–72]. The mechanisms of the lifespan-prolonging activity of ER in* Drosophila* were widely investigated at molecular levels. Up to date, the most recognized mechanisms for ER are related to its effect on the metabolic rate, the nutrient sensing insulin/IGF-1 like pathway, the TOR pathway, apoptotic pathway, sirtuin pathway, and olfactory and gustatory system [73]. In addition, ER has been proposed to be associated with lesser damage of cellular macromolecules such as DNA [74], proteins [75], and lipids [76].

We have studied the gene expression of SOD1, SOD2, CAT, Rpn11, and Mth in fruit flies fed one of the three diets, namely, energy restriction diet (ER, 0.39 kcal/mL diet), standard energy diet (SE, 0.78 kcal/mL diet), and high energy diet (HE, 2.35 kcal/mL diet). Results showed that ER increased the mean lifespan by 16% compared with the control flies. It was demonstrated that ER group had a greater activity and gene expression of SOD1 and SOD2 than other two groups of flies. The elevated expression of Rpn11 induced by ER was observed at some time points, suggesting that the interaction of ER with Rpn11 may also mediate the lifespan-prolonging activity of ER. However, ER had no effect on the gene expression of CAT and Mth. The lifespan prolonging activity of ER was at least partially mediated by its effect on gene expression of SOD and possible Rpn11 but unlikely on the gene expression of CAT and Mth. It is also possible that the lifespan prolonging activity of ER is not due to its effect on a single gene rather than on a cluster of genes involved in oxidative stress, IIS pathway, apoptotic pathway, programmed autophagy, and the olfactory system.

## 5. Antiageing Nutraceuticals and Functional Foods

The term “nutraceutical” is actually a combined form of “nutrition” and “pharmaceutical.” The generally accepted definition is “a food or part of a food which provides health benefits, including the prevention and/or treatment of a disease.” Most nutraceuticals are dietary supplements. Studies both in vitro and in vivo reveal that consumption of nutraceuticals, especially the ones with high antioxidant capacity, has an inverse relationship with cardiovascular diseases, various cancers, and diabetes. However, their antiageing activity is yet to be proven. On the basis of FRTA, it is postulated that any substance with a great antioxidant capacity can be a potential candidate for delaying the aging.

### 5.1. Tea Catechins and Theaflavins

**Figure 2:**
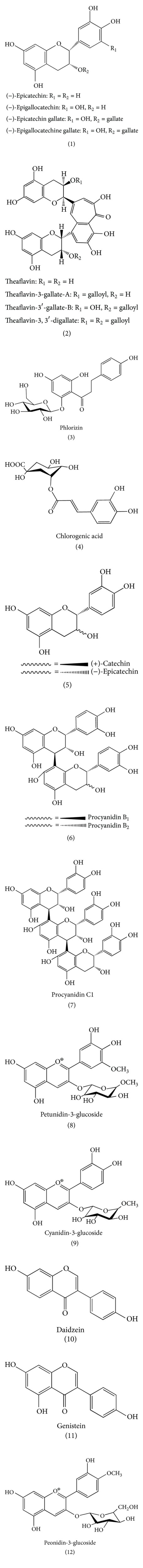
Chemical structures of (1) green tea catechins, (2) black tea theaflavins, (3–7) polyphenols in apple, (8, 9) blueberry anthocyanins, (10, 11) soybean isoflavones, and (9, 12) black rice anthocyanins.

Tea, next to water, is the second most popular beverage consumed by humans in the world. Black tea is more widely consumed in Western countries while green tea is preferred in the Eastern world. Black tea extracts mainly contain catechins and theaflavins (Figure 2). Evidences from clinical trials suggest that consumption of tea has various health benefits. Leenen et al. [77] demonstrated that drinking either green tea or black tea would lead a significant increase in plasma antioxidant potential by ferric-reducing antioxidant power (FRAP) assay. Furthermore, it has been reported in different population studies that consumption of green tea or black tea could significantly reduce DNA oxidation and lipid peroxidation [78, 79].

As to the antiaging activity of tea, previous studies conducted in this laboratory revealed that green catechins and black theaflavins could extend mean lifespan of* Drosophila* by 10–16%. This was accompanied by greater expression of the endogenous antioxidant enzymes SOD and CAT [60, 61, 80] (Table 2). Studies on* C. elegans* also showed similar results, indicating that treatment of epigallocatechin gallate (EGCG), an active ingredient in tea, would lead to a significant longer lifetime [81, 82]. In mice, consuming tea polyphenol, starting from 13 month till death, could increase the average lifespan by more than 6% [83].

### 5.2. Apple Polyphenols

A proverb says “one apple a day keeps doctors away.” Apple has been recognized as a healthy fruit in many cultures. It contains a large number of phytochemicals, mainly polyphenols with strong antioxidant activities, including chlorogenic acid, phloretin, proanthocyanidin B2, epicatechin, catechin, and rutin (Figure 2).

Consumption of apple has been inversely associated with the risk of cardiovascular disease, hypercholesterolaemia, and various cancers. The Women's Health Study, involving almost 40,000 women with a 6.9-year follow-up, examined the correlation between flavonoids and cardiovascular disease, finding an inverse correlation between cardiovascular disease and consumption of apples [84]. The Iowa Women Study on nearly 35,000 women revealed that apple consumption was inversely related to the death caused by coronary heart diseases in postmenopausal women [85]. Furthermore, several clinical studies have linked apple consumption with a lower risk of cancers, especially lung cancer. It was found that eating apples would reduce the risk of lung cancer, with being more effective in women than in men [86, 87].

Experiments on animals showed similar results and revealed some potential mechanisms of the beneficial effects of apple. It was reported that, in cholesterol-fed rats, there was a significant reduction of plasma and liver cholesterol level along with increased amount of high density lipoproteins (HDL) [88]. Another study conducted by Leontowicz et al. [89] has demonstrated that apples have much better cholesterol lowering effects than pears and peaches, suggesting that, having similar amount of fiber content, apples' superior activity might be due to its larger quantity of phenolic components. Apple has been proved effective in inhibiting low-density lipoprotein (LDL) oxidation while the greatest inhibitory effect comes from apple peels [90]. In addition, apple can greatly inhibit the growth and proliferation of liver and colon cancer cells [91, 92]. Moreover, apple juice concentrate has been demonstrated to be effective in neuroprotection in both genetically compromised and normal aged mice [93–95]. However, antiageing activity of apple and the underlying mechanisms remain elusive.

We have studied the effect of apple polyphenols (AP) on the lifespan of fruit flies and its interaction with gene expressions of SOD, CAT, Mth, Rpn11, CcO subunits III, and VIb [96]. Results showed that AP could extend the mean lifespan by 10% in fruit flies. This was accompanied by upregulation of gene SOD1, SOD2, and CAT while downregulation of Mth in the aged fruit flies. Chronic paraquat exposure could shorten the maximum lifespan from 68 to 31 days and reduce the climbing ability by 60%, while supplementation of AP into diet could partially reverse the paraquat-induced mortality and decline in climbing ability. AP could upregulate Rpn11 while it appeared to have no significant effect on gene expression of ubiquitinated protein, CcO subunits III and VIb. It was therefore concluded that the antiaging activity of AP was, at least in part, mediated by its interaction with genes SOD, CAT, Mth, and Rpn11 [96].

### 5.3. Blueberry Extracts

Blueberries, containing large amounts of polyphenols, possess a greater antioxidant capacity than most other fruits and vegetables [103, Figure 2]. It has been reported that consumption of natural compounds in blueberries can retard the age-related physiological and functional deficits [104]. Krikorian et al. [105] have evaluated the health benefits of blueberry supplementation, revealing that daily consumption of wild blueberry juice for 12 weeks would improve memory function in older adults with early memory decline. However, larger sample size and more consistent clinical data are lacking to draw a conclusion.

Studies in vitro and in vivo on experimental animal models also provide solid and inspiring results. Galli et al. [106] claimed that blueberry supplemented diet could reverse age-related decline in hippocampal heat shock protein (HSP) in rats. Similarly, blueberries are also suggested effective in enhancing cognitive and motor behavior as well as attenuating cognitive declines in object recognition memory in aged rats [107]. Furthermore, age-related deficits in NMDAR-dependent long-term potentiation, a cellular substrate for learning and memory, are also reported to be ameliorated by blueberry enriched diet [108].

We have investigated the lifespan-prolonging activity of blueberry extracts in fruit flies and explored its underlying mechanism. Results revealed that blueberry extracts at 5 mg/mL in diet could significantly extend the mean lifespan of fruit flies by 10% [97]. Result was in agreement with that of Wilson et al. [109], who demonstrated that blueberry extract, mainly the fraction enriched in proanthocyanidin compounds, in diet could increase lifespan and slow ageing related declines in* C*.* elegans*. In our study, it was found that the mean lifespan extension was accompanied by upregulating gene expression of SOD, CAT, and Rpn11 and downregulating Mth gene [97]. Intensive H_2_O_2_ and paraquat challenge tests showed that lifespan was only extended in Oregon-R wild type flies but not in* SOD* ^***n108***^ (deficiencyin SOD) or* Cat* ^***n1***^ (deficiency in Cat) mutant strains, indicating that the prolongevity activity of blueberry was mediated by its enhancement on endogenous antioxidant system. Chronic paraquat exposure shortened the maximum survival time from 73 to 35 days and decreased the climbing ability by 60% while blueberry extracts at 5 mg/mL in diet could significantly increase the survival rate and partially restore the climbing ability with upregulating SOD, CAT, and Rpn11. It is clear that blueberry extract could affect the gene expression of Mth, Rpn11, and endogenous antioxidant enzymes SOD and CAT, thus leading to the mean lifespan extension (Table 2).

### 5.4. Soybean Isoflavones

Soybeans are considered as a great source of complete protein, which contains all the essential amino acids in sufficient amounts for human use [110]. They can serve as a good alternative to animal proteins for vegetarians. Daidzein and genistein, the main isoflavones in soybeans, possess the antioxidant activity.

The notion that consumption of soy protein could offer health benefits has been popular during the past decades. Soy protein in diet has been inversely associated with hypercholesterolaemia, bone loss, and various cancers. According to Food and Drug Administration (FDA), “25 grams of soy protein a day, as part of a diet low in saturated fat and cholesterol, may reduce the risk of heart disease.” The meta-analysis conducted by Anderson et al. [111, 112] demonstrates that consumption of soy protein can decrease serum total cholesterol, LDL cholesterol, and triacylglycerol concentrations. Meanwhile, it is claimed that the decreasing effect is at least partially related to subjects' initial cholesterol concentrations and isoflavones might account for at least 60% of the cholesterol-lowering effects of soy protein [111]. More than 50 trials since then, investigating health benefits of isoflavones, have been conducted [113, 114]. It has been further demonstrated that LDL reduction induced by soy protein without isoflavones is mild, indicating that isoflavones might be the main active compounds, contributing to the cholesterol-lowering effects [115, 116]. Besides that, evidences from clinical studies reveal that consumption of soy foods, especially isoflavones, leads to higher femoral/lumbar spine bone mineral density in postmenopausal women [117]. It is also reported that in Asian countries where soy foods are more prevalent, the incidence of breast and endometrial cancer is relatively low. Actually plasma genistein in Japanese can reach 4 *μ*M while the one can be as low as 40 nM in people consuming a typical western diet [118, 119]. Moreover, the case-control studies carried out by Shu et al. [120] and Wu et al. [121] have proved that high amount of soy intake are associated with low risk for breast cancer. However, epidemiological findings on its anticancer activity are not as consistent as the ones on its cholesterol-lowering effect.

Though the underlying mechanisms for the efficacy of soybean isoflavones are still not fully understood, studies on cells, isolated arteries, and animals provide insightful clues. It is stated that isoflavones are able to activate endothelial nitric oxide synthase, exerting vasodilatory effect [118]. Moreover, studies on isoflavones' effects on vascular smooth muscle cells (VSMC) reveal that isoflavones can inhibit cell proliferation and DNA synthesis [122]. Generally speaking, it is believed that actions of isoflavones largely overlap with those of estrogens, especially for its influence on cardiovascular diseases [123].

We have investigated the soybean isoflavones extract on the mean lifespan and expression levels of genes SOD, CAT, and Mth in fruit flies. Results demonstrated that soybean isoflavones extract in diet could significantly increase mean life span of fruit flies with upregulation of endogenous antioxidants SOD1, SOD2, and CAT on both mRNA and protein level in selected time points with no effect on MTH (unpublished data). Result was in agreement with that of Borrás et al. [124], who showed that antioxidant activity of genistein was mediated via the upregulation of antioxidant gene expression, such as increased mRNA levels of MnSOD and activation of NF*κ*B, suggesting that supplementation of isoflavones may be beneficial in decreasing oxidative stress, thus contributing to lifespan extension. However, Altun et al. [125] recently found that genistein would decrease the maximum lifespan of female* D. melanogaster*.

### 5.5. Black Rice Anthocyanins

Black rice is an excellent source of dietary antioxidants. It is widely consumed in China. Supplementation of black rice confers some health benefits including anticancer, anti-inflammation, antidiabetes, and anti-Alzheimer's disease. Composition analysis shows that black rice is rich in fiber, vitamin E, and polyphenols. The antioxidant activity of black rice is mainly ascribed to the high content of anthocyanins, two majors of which are cyanidin-3-O-glucoside and peonidin-3-glucoside [126], with cyanidin-3-O-glucoside accounting for more than 80% of total anthocyanins [127, Figure 2]. Wang et al. [128] compared the antioxidant capacities of 14 anthocyanins using the automated oxygen radical absorbance capacity (ORAC) assay, and the result showed that cyanidin-3-O-glucoside has the highest ORAC activity, which was 3.5 times stronger than Trolox (vitamin E analogue). The further indepth insight into the antioxidative mechanism of black rice demonstrated that anthocyanins were inhibitors of xanthine oxidase, one of the generators of superoxide anion radicals [129].

We have investigated the lifespan-prolonging activity of black rice anthocyanins extracts and its effect on gene expressions of SOD1, SOD2, CAT, Mth, and Rpn11 [98]. Results demonstrated that black rice anthocyanins at 30 mg/dL could prolong the mean lifespan of fruit lies by 14% accompanied with upregulation of mRNA SOD1, SOD2, CAT, and Rpn11 and with downregulation of Mth. In addition, black rice anthocyanins at 30 mg/dL increased the survival time of Alzheimer transgenic line A*β*42 33769 with chronic exposure to paraquat. Huang et al. [130] found that black rice possessed antiaging, antihypoxia and, antifatigue effects in subacute ageing model mice.

## 6. Conclusion

Many natural antioxidants, nutraceuticals, and functional foods have been identified as free radical or active oxygen scavengers. Functional foods and nutraceuticals which possess the antioxidant activity may play an important role in delaying the aging (Table 2). Development and research on these functional foods and nutraceuticals are of interest to both public and scientific community. To better understand their antiaging activity, it is essential to identify the active ingredients and underlying mechanisms. On the basis of limited research, it appears that dietary antioxidants have the antiageing activity at least in fruit fly model, most likely by enhancing endogenous enzymatic defense capacity via upregulation of SOD and catalase and suppression on formation of free radicals.

## Figures and Tables

**Table 1:** Selected longevity determined genes recently recognized in fruit flies, for which allelic variation is associated with extension in longevity.

Gene names	Molecular mechanism
*Mth *	A P-element insertion at *Mth* increases lifespan by 35% [31]
*Indy *	P-element insertion at *Indy* shows extended mean lifespan [35]
*Chico *	Heterozygous for *chico* shows an increase in median lifespan [36]
*dTOR *	Inhibition of TOR pathway leads to 24–26% lifespan extension [37]
*Sirtuins *	Gene upregulation of sirtuins increases the lifespan [38]

**Table 2:** Effect of selected nutraceuticals or functional foods on ageing and the possible underlying mechanisms.

Phytochemical antioxidants	Dose	Mean lifespan extension	Molecular mechanism	Reference
Apple polyphenols	10 mg/mL	10%	Upregulate SOD1, SOD2, Cat, and Rpn11 genes. Downregulate MTH gene	[96]
Blueberry anthocyanin extract	5 mg/mL	10%	Upregulate SOD1, SOD2, Cat, and Rpn11. Downregulate MTH gene	[97]
Black rice anthocyanin extract	30 mg/mL	14%	Upregulate SOD1, SOD2, Cat, and Rpn11 genes. Downregulate MTH gene	[98]
Green tea catechin extract	10 mg/mL	16%	Upregulate CuZnSOD, MnSOD, and Cat genes	[60, 61]
Black tea theaflavins	5 mg/mL	10%	Increase CAT activity. Upregulate SOD1 and Cat genes	[80]
Sesamin	2 mg/mL	12%	Upregulate SOD1, SOD2, and Rpn11 genes.	[99]
Curcumin	100 *μ*M	19%	Downregulate the expression of several aging-related genes, including TOR*, InR, Hep*, *sun*, and *mth *	[100]
Marine microalga DHA-rich extract	10 mg/mL	10%	Upregulate SOD1 and SOD2 genes. Downregulate MTH gene	[101]
Nectarine extract	4%	14–22%	Reduce the transcript level of phosphoenolpyruvate carboxykinase (*PEPCK*), iron regulatory protein 1B (*Irp-1B*), *4E-BP*. Influence the redox status and reduce oxidative damage indirectly through modulate the JNK signaling pathway.	[102]
